# Procedural Control Versus Resources as Potential Origins of Human Hyper Selectivity

**DOI:** 10.3389/fpsyg.2021.718141

**Published:** 2021-07-26

**Authors:** Ulrich Ansorge, Christian Büsel, Marlene Forstinger, Daniel Gugerell, Markus Grüner, Ulrich Pomper, Moritz Stolte, Rebecca Rosa Schmid, Christian Valuch

**Affiliations:** ^1^Department of Cognition, Emotion, and Methods in Psychology, University of Vienna, Vienna, Austria; ^2^Vienna Cognitive Science Hub, University of Vienna, Vienna, Austria; ^3^Research Platform Mediatised Lifeworlds, University of Vienna, Vienna, Austria; ^4^Department of Psychology, University of Innsbruck, Innsbruck, Austria; ^5^Georg-Elias-Müller-Institut für Psychologie, University of Göttingen, Göttingen, Germany

**Keywords:** procedures, resources, cueing, Simon effect, dual-process (dual-system) models

## Abstract

In the current review, we argue that experimental results usually interpreted as evidence for cognitive resource limitations could also reflect functional necessities of human information processing. First, we point out that selective processing of only specific features, objects, or locations at each moment in time allows humans to monitor the success and failure of their own overt actions and covert cognitive procedures. We then proceed to show how certain instances of selectivity are at odds with commonly assumed resource limitations. Next, we discuss examples of seemingly automatic, resource-free processing that challenge the resource view but can be easily understood from the functional perspective of monitoring cognitive procedures. Finally, we suggest that neurophysiological data supporting resource limitations might actually reflect mechanisms of how procedural control is implemented in the brain.

## Introduction

In the current review, we highlight that, in empirical research on cognitive resources, it is important to understand the specific reasons for the selectivity of human information processing before drawing conclusions about limited resources as the cause of such selectivity. We argue that many cases of selectivity reflect functional benefits rather than structural constraints. From the perspective of an updated selection-for-action view, we remind the reader that selectivity in human information processing is often functional rather than structural: it is often the consequence of an intentional restraint to focus on the most important information rather than a reflection of limited cognitive resources. Think of top-down search for a color-defined target, for instance, for your red suitcase on a baggage belt. Here, it is necessary to facilitate the processing of red colored objects relative to other objects of a different color. The reason for this type of selectivity is not limited resources in the sense of a time-invariant structural constraint. Instead, this selectivity serves a purpose and reflects a functional constraint that could vary over time, depending on what is intended and required by the task. In a different situation, it might be helpful to search for a different feature than red, such as when I look for my blue socks in a drawer. Functional selectivity can also take on additional limitations, for example, resulting in a tight focus on a single feature even where resource estimates would allow selection and processing of more features. Importantly, humans are typically concerned with some type of intentional, goal-oriented information processing. Following the pick-up of my suitcase at the airport, for example, I would next have to find the exit, navigate my way to my rental car booth, etc. As these examples show, functional selectivity in purposeful and goal-oriented behavior is abundant.

**FIGURE 1:**
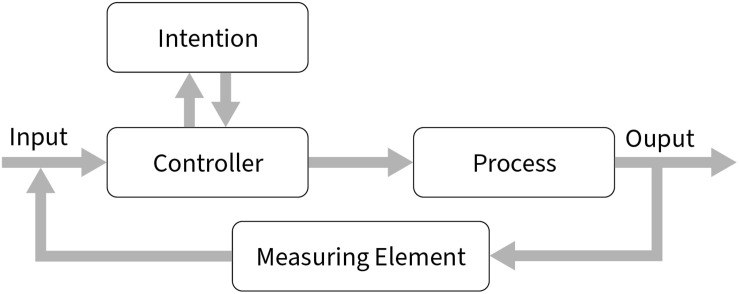
Procedural control in a closed-loop system works similarly to any feedback loop. The input (e.g., a text) would be checked for fitting content by the controller (i.e., a steering value determined by, e.g., an intention to search for errors in a list of references) to be processed (e.g., read and correct references) until a measuring element signals that the purpose is fulfilled (e.g., no further errors could be found in a list).

Importantly, we suggest that what applies to actions also applies to cognitive procedures in general, whether they result in overt behavior or not. In this context, procedures are the top-down controlled cognitive processes that humans conduct with a particular purpose or intention in mind (for a general architecture, see Figure 1). Thus, an updated selection-for-action view is better denoted a selection-for-procedures view. This perspective generalizes the distinction between functional and structural causes of selectivity from action control to the control of other vital cognitive processes lacking any obvious action correlates such as (latent) learning, reasoning, problem solving, comprehension, or the encoding and retrieval of knowledge.

At the outset, we sketch how top-down control of procedures is responsible for attention in its broadest sense: selectivity of human information processing. Next, we will give examples of hyper selectivity – how human information processing sometimes appears more selective than would be expected based on capacity limitations alone – and discuss how the selection-for-procedures view explains this discrepancy. In the course of our argument, we critically review dual-process theories of resource-demanding versus resource-free processing and, finally, point out how arguments for neuronal resources as the ultimate cause of selectivity in human information processing fall short of ruling out the selection-for-procedures view.

## Selectivity From a Functional Perspective

When humans are confronted with several cognitive tasks at the same time, their performance is typically lower in terms of accuracy or speed than under single-task conditions (e.g., Navon and Miller, 1987; Pashler, 1994), and it takes time to switch between tasks (Rogers and Monsell, 1995). These observations laid the ground for the assumption that human information processing depends on limited resources (cf. Kahneman, 1973; Navon and Gopher, 1979; Wickens, 1980, 1984).

However, we believe that all too often researchers jump to this conclusion without properly considering alternative interpretations of this basic finding. The rationale is that if only one task could be performed at a time, a capacity limit must have hampered performance of both tasks; since what should be wrong with solving more tasks at a time if this were possible? However, in our view, caution is advised in drawing this conclusion, as there is one alternative interpretation of the findings that should not be dismissed easily (cf. Navon, 1984). Selectivity could result from an intentional, functional limitation by the human agent rather than simply a structural resource limitation imposed upon human performance. According to this functional view, it is not a structural (i.e., time- and situation-invariant) limitation that causes selectivity. Instead, selectivity results from the fact that humans, often without noticing, focus on the most important aspects for controlling their actions and information processing in general. Which information is selected for prioritized processing ultimately depends both on human agent’s current goals and prior experiences that have taught them how to efficiently complete similar tasks.

This has been emphasized, for example, in the selection-for-action view (Allport, 1987; Neumann, 1987). The selection-for-action view stresses that the necessity to carry out actions in time requires that top-down monitored information is processed continuously, so that information selection is optimally synchronized with the executed action as it unfolds. For example, think of the changing spatial input of a moving object you seek to keep track of via smooth pursuit eye movement. Another major emphasis of the selection-for-action view is that actions serve intended purposes that need to be top-down monitored for successful execution (cf. Lotze, 1852; von Holst and Mittelstaedt, 1950; Miller et al., 1960; Greenwald, 1972; Blakemore et al., 1998; Botvinick et al., 2001; Franklin and Wolpert, 2011; Janczyk and Kunde, 2020). Prioritized monitoring of the most important steering values and disregard for less important information is the major contender to a limited resources explanation of selectivity in human information processing (cf. Dreisbach et al., 2007; Dreisbach, 2012). This is especially true, where the number of currently top-down monitored features or objects is lower than would be expected based on known capacity limitations (cf. Eitam et al., 2013).

In fact, the human inclination to focus on the most relevant information – that is, information related to the success (or failure) of action goals – is not only a characteristic of overt actions but actually of any type of top-down controlled mental procedure, including those that do not manifest in overt behavior (cf. Anderson et al., 2004; see also Figure 1). As an example, consider the solution for a categorical syllogism. For a valid conclusion, humans have to select the major term of the major premise (e.g., “all bees are *insects*,” with the major term in italics) and the minor term of the minor premise (“a *bumblebee* is a bee,” with the minor term in italics). To draw a valid conclusion (such as “bumblebees are insects”), they would have to compare major and minor terms with the middle term (here, “bee”). Importantly, each step toward a proper conclusion – successful selection of each term in turn, plus the comparison at the end – must be monitored. That is, the steering value for monitoring covert processing would have to shift along with the successive steps of the operation. It might be argued that the first two selections could be conducted in parallel. However, this is unlikely, as typically the premises would be read, heard, or remembered in a phonological and, thus, sequential process. More importantly, this example shows that some of the selections require focusing on one particular feature or object, here, a term. Otherwise, errors would follow suit. For instance, mixing up the selection order of the minor term and the comparison would mean that the cognitive procedure – with the goal of a valid conclusion – would ultimately not be monitored appropriately for its success. This example of a sequentially unfolding procedure illustrates that sequential selections are often an inevitable constraint in the cognitive processing of meaningfully related information.

In addition, even where two features or two sources of information could be covertly processed in parallel, it is always possible to willingly focus on only one of them at a time if simply for reasons such as stopping short of a true capacity limitation (and, thus, not risking running into a capacity limitation, i.e., risk avoidance), endowing a mere content-wise “topical” difference between processed features or information with a redundant discriminating temporal tag within the processing sequence, or simply as a result of overgeneralization of sequential processing from situations where sequential processing is necessary to situations where it is not. In other words, “additional” cognitive control beyond what would be currently required to solve a task is not only associated with costs but also with value (cf. Dreisbach, 2012; Shenhav et al., 2017).

The general idea of closed-loop information processing also gained traction in theories of perception. Think of reentrant processing (Di Lollo et al., 2000; Pascual-Leone and Walsh, 2001), predictive coding (Friston and Kiebel, 2009; Clark, 2013; Press et al., 2020), or the sensorimotor hypothesis of vision (O’Regan and Noë, 2001). All of these theories share the central tenet that a past state of the cognitive system (e.g., a sensory activation, a memory trace, a motor command) is compared with a current state. Typically, this is done for purposes akin to monitoring or updating, such as deriving an “error” or “deviation” estimate (between initial and current state; Friston and Kiebel, 2009; De Lange et al., 2018), a refreshed impression (Di Lollo et al., 2000), or a particular qualitative experience (e.g., of seeing a particular color; O’Regan and Noë, 2001). Take the example of Bar’s (2007) proactive-brain hypothesis: during visual recognition, an initial sensory state of low-spatial frequency information serves as a hypothesis, reducing the number of possible candidate objects for recognition through activation of potentially fitting templates in memory. In a subsequent step, more fine-grained high-spatial frequency information either confirms or revokes the initial hypothesis (or activated template) and, thus, objects are perceived more or less efficiently, respectively. Importantly, the function of such monitoring (e.g., of prediction, of gaining an error signal, of correction of an initial state, of experiencing a specific perceptual quality) would not be achieved if just any information would be selected for comparison. Instead, in all of these theories, functions are only served if past and current information are related to one another. Thus, selectivity, the human ability to prioritize some information – features, locations, “channels,” modalities, or tasks – is an inevitable consequence of many, if not all, action, perception, and cognitive procedures serving an intentional goal. From this perspective, it appears grossly negligent to consider evidence of selectivity generally as proof for limited cognitive resources (cf. Duncan, 1980; Navon, 1984).

Skeptics might want to interject that these particular forms of selectivity could merely reflect information accumulation across time: for instance, more evidence supporting a particular prediction where past and, thus, “expected” inputs are more similar to one another than where they differ. This view, however, fails to account for the fact that goals or purposes are decisive for the “fate” of information accumulated across time. For example, while repeated visual input sometimes facilitates selection as in priming of visual attention (cf. Maljkovic and Nakayama, 1994; Kristjánsson and Campana, 2010; Valuch et al., 2017), humans also show the opposite tendency in other situations – that is, a preference for the selection of novel input that deviates the most from what is expected or what has been seen (Horstmann, 2002, 2005; Itti and Baldi, 2009; for a discussion of the principles in action control, see also Feldman and Friston, 2010; Jiang et al., 2013; Press et al., 2020). Whether repeated or novel information is selected for processing could, in many cases, depend on the requirements of the task at hand (cf. Müller et al., 2009; Gaspelin and Luck, 2018). Thus, framing perceptual selection in the context of purposeful and expectancy-based procedures allows understanding this malleable and flexible nature of relating past to present input. In contrast, a simple accumulation of input across time will not do.

## Examples of Selectivity That Challenge a Limited Resources Explanation

Are there any criteria that decide if a given case of selectivity reflects functional or structural constraints (see also Box 1)? This is indeed a thorny issue, and we are not certain that any criterion will be entirely convincing. In the following, however, we provide two simple examples that demonstrate hyper selectivity at variance with assumed resource capacity limitations: the flanker effect and switch costs associated with searching for two colors instead of a single one. Both instances are unexpected examples of hyper selectivity that is stronger than what would be expected on the basis of the assumption of limited resources, as the tasks impose seemingly low processing demands. From these examples, we derive general insights that might be of use for deciding if observed selectivity is due to functional or structural (i.e., resource limitations) constraints.

**Box 1.**

There are some cases that challenge both the concept of resource limitations, and our notion of functional selectivity. While we explore evidence in favor of functional selectivity more in depth in the main text, we did not want to leave out conflicting evidence, which we mention here.

To prove resource theory wrong, some researchers sought to falsify selectivity and demonstrate processing abilities free of resource limitations under appropriate training or instruction conditions (cf. Allport et al., 1972). An impressive example is Schumacher et al.’s (2001) falsification of the “central bottleneck” (as a limiting resource that could only be used for one task at a time) or the resulting “psychological refractory period effect” – that is, the cost of performing two tasks simultaneously as compared to the same tasks alone (Pashler, 1994). Take a second example. While research on visual working memory suggests an upper capacity limit concerning how many objects can be remembered and reproduced from a memory set (e.g., Luck and Vogel, 1997; Cowan, 2010), other tasks suggest that humans can effortlessly surpass this limitation and represent perceptual information from large crowds of objects, well beyond what would be expected based on the suggested memory resource limitations. This is illustrated in the phenomenon of ensemble perception (Ariely, 2001; Alvarez and Oliva, 2008) which can be observed with tasks that do not require remembering and reproducing each object from a group individually but rather assessing summary characteristics of the group, such as the mean and range of features present across many object exemplars. Ensemble perception has been reported for relatively simple feature dimensions such as size, orientation, color, or motion direction but also more complex characteristics such as the gender or emotional expression of faces, or the apparent lifelikeness of objects (Whitney and Yamanashi Leib, 2018). Such results are not easy to explain from the perspective of limited processing resources. Thus, rather than reflecting limited processing or representational resources, the observed upper bound in explicit working memory capacity could stem from specific task requirements and the way that processing is probed at the end of each experimental trial. As a consequence, using the very same object arrays as stimuli, one could reach very different conclusions about capacity limitations, depending on how cognitive processing is assessed.

Given what we have argued for above – the functionality of selectivity, the benefits of concentrating on one steering value at a time – these findings are not entirely in line with the predictions of a selection-for-procedures view either. Therefore, we take the opposite perspective and point out two instances of unexpected hyper selectivity that is stronger than what would be expected on the basis of the assumption of limited resources, as the tasks impose seemingly low processing demands.

As a first example, we turn to flanker interference (Eriksen and Eriksen, 1974; Gratton et al., 1988; but see Franconeri, 2013). In the flanker task, one can observe increased interference between alternative letters – a central target letter and one or several peripheral flanking letters – simply by assigning alternative responses to the different letters (cf. Botella, 1996): compared to a response-irrelevant condition, in which only one of two letters, say an *A* as a target presented together with a *T* as a flanker, requires a response, reaction times increase in a response-incongruent condition for responses to the same target letter *A*, now presented in the context of a flanker letter *T* that would require a different response if used as a target in another trial. Interestingly, increased response times under incongruent conditions are even reliably observed if the two alternative responses have to be given with the index fingers of the left and right hands, respectively (Gratton et al., 1988, 1992). This is puzzling, as it is, of course, possible to give responses with the two hands almost simultaneously (e.g., Mechsner et al., 2001). Think of pressing two keys on a piano simultaneously. How can it be that a simple instruction to use the two fingers to indicate different stimuli transform two commensurable (i.e., simultaneously executable) actions into alternatives that create a cost when activated at the same time? In our view, this is only possible if humans represent the corresponding actions intentionally as alternatives, which, in turn, requires monitoring whether the conditions for each of these alternatives are met. In other words, humans have to set up top-down control representations to twist “parallel processing” of motor program execution artificially into a sequential procedure of allowing the use of either one or the other finger. To note, this type of interference by assigning alternative responses to the letters is not the same as the psychological refractory period (cf. Welford, 1952; Pashler, 1994). The latter suggests that a decision in a Task *A* blocks a decision in a Task *B* until the decision in task *A* has been made. In contrast, interference by defining mutually commensurable responses as alternatives is more like creating the critical preconditions of a decision in a task in the first place. To note, however, the resulting cost of representation of responses as alternatives exceeds that of the decision itself. Botella (1996) showed that a decision between one response-associated target letter and an alternative “no-go” distractor, which was not associated with any response, created a cost and, thus, maybe a psychological-refractory period effect. However, this effect was substantially smaller than the interference by a response-incongruent flanker stimulus.

**FIGURE 2:**
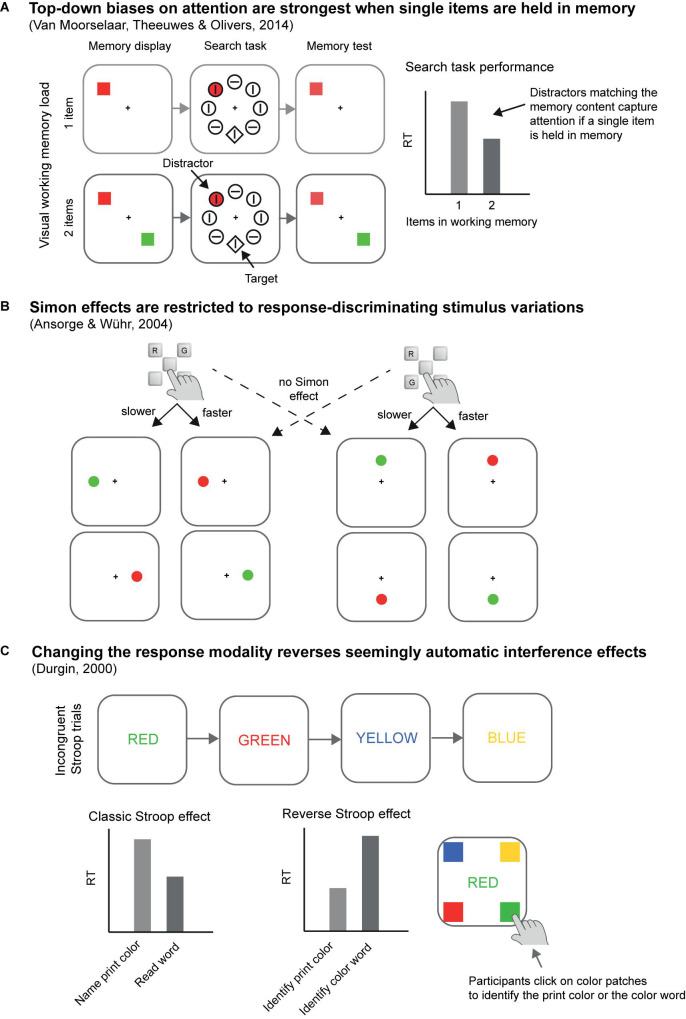
Examples of empirical results that challenge a rigid limited resources view. **(A)** Top-down biases on attention are strongest when single items are held in memory. Related to the results of Büsel et al. (2019) described in the main text, the results of Van Moorselaar et al. (2014) illustrate that attention is captured by distractors that match the working memory content only if a single color is kept in memory, but this capture effect already vanishes if two colors are kept in working memory, even though this should not exceed generally assumed capacity limits. **(B)** Ansorge and Wühr (2004) found out that Simon effects are restricted to response-discriminating stimulus variations. The key mapping, that is, whether the alternative response keys for red (R) or green (G) stimuli were arranged in a horizontal or a vertical configuration varied between participants and red and green target stimuli occurred either along the horizontal or vertical meridian. Crucially, spatial stimulus-response compatibility effects (Simon effects) – facilitation for responses that shared location codes with targets (e.g., right responses to right targets) relative to responses and targets of different locations (e.g., right responses to left targets) – occurred only in those conditions where the axis of stimulus variations corresponded with the spatial response axis. The same compatibility effects were missing with regards to the non-varying spatial response axis, suggesting that location selection reflected response monitoring rather than response execution. **(C)** Changing the response modality reverses seemingly automatic interference effects. Durgin (2000) reversed the Stroop effect simply by asking participants to click on color patches corresponding to the word meaning rather than utter the print color names. For further discussion see main text.

A second striking example comes from our own research where we found that asking participants to search for two instead of a single color in a visual search task incurred a processing cost (Büsel et al., 2019). Compared to a single-color block, in which participants had to search for one color-defined (e.g., red) target among differently colored distractors, dual-color blocks, where participants searched for two possible target colors (e.g., red or green) while presenting only one of these per trial, produced switching costs and mixing costs (cf. Kiesel et al., 2010). Here, switching costs mean that changing the target color from one trial to the next slowed target search compared to repeating target color in consecutive trials. Mixing costs mean that in dual-color blocks target search in target-color repeat trials was slower than in single-color blocks. The results suggest continued usage of a top-down search template for a specific color (e.g., a search template for red targets) in the dual-color blocks, just as if participants preferentially only searched for a single color at a time rather than for both colors simultaneously (see Box 2). Related to these findings, Van Moorselaar et al. (2014) reported that colored distractors that match an item held in visual working memory only capture attention in conditions where participants keep a single colored item in working memory but not when two items are held in working memory (see Figure 2A).

**Box 2.**

Here, we describe in brief a reanalysis of data originally published by Büsel et al. (2019). The purpose of this reanalysis was to investigate whether participants showed a preference for one of two colors while engaging in dual-color search. Presenting a non-predictive cue prior to the target display in a visual search experiment can facilitate target search with cues at target position (valid condition) relative to cues presented away from the target (invalid condition), especially or even selectively if the cue matches the search template for the targets (Folk et al., 1992; Folk and Remington, 1998). For instance, during search for red targets, a red but not a green cue would lead to a validity effect - with faster search in valid than invalid conditions - reflecting attention capture by the non-predictive cue (such that attention would be at target position from target onset in valid but not invalid conditions). During search for two target colors, we observed that only a single color was used as a search template at a time (Büsel et al., 2019). In the present textbox, we tested a novel hypothesis regarding the origin of this selectivity. If single-color search (e.g., for green targets) in one block before two-color search (e.g., for red and green targets) in a second block suggests to the participants a preference for the usage of the color used in both blocks (e.g., green), we expected to find more capture by top-down matching cues with a color used for targets in both blocks (e.g., green) than by top-down matching cues with a color used for targets in the two-color search block only (e.g., red).

**Method**

*Participants*. In total, 68 participants completed the experiment in Büsel et al. (2019).

*Design and procedure*. Participants were asked to complete four experimental blocks: two blocks in a single target-color version of the cueing task and two blocks in a dual target-color version of the same task. In single target-color blocks, the target was either always red or always green. The target-preceding cues could either match the searched for color (e.g., searching for a green target preceded by a green cue) or not (blue cue). In the dual target-color blocks, the target-color could randomly be either red or green. Consequently, preceding cues that were red or green matched the searched-for colors, whereas, again, blue colors did not match the task-relevant colors.

**FIGURE 3:**
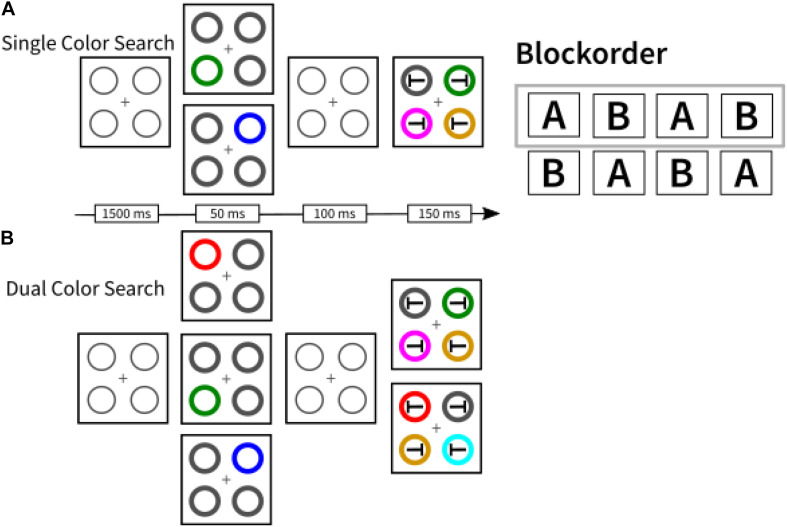
A schematic depiction of the two search conditions used in Büsel et al. (2019). In single color search blocks **(A)**, participants were required to search for the same target feature throughout the whole block (here, for example, green). In dual color search blocks **(B)**, participants searched for a target that could randomly either be red or green. Block order was balanced across participants. For the analysis presented in Box 2, we used data from participants in the A-B-A-B block order condition (framed gray).

Participants’ task was to report the orientation of the ‘T’ embedded within the circle carrying the target-color. The block order was balanced across participants and could be either A-B-A-B or B-A-B-A (here: A = dual; B = single; see Figure 3).

**Analyses**

In order to have a sufficient number of measurements per participant, we only analyzed participants in the A-B-A-B block order (*N* = 32). With these participants, we performed a repeated-measures analysis of variance (ANOVA), with the factors validity (valid, invalid) and whether the presented cue had the same color as the relevant color in the preceding single-color search block (yes, no). Non-matching cues were excluded from this analysis.

*Response times*. The interaction between both variables was significant, with *F*(1,31) = 10.99, *p* < 0.01, $\eta_{\text{p}}^{2}$ = 0.26. *Post hoc* paired *t*-tests revealed significant validity effects by cues that shared features with the previously relevant target-color in single-color search blocks, 22 ms, *t*(31) = 3.43, *p* < 0.01, *d* = 0.31. Conversely, top-down matching cues carrying features that were previously irrelevant even led to an inverted, albeit not significant, validity effect of −10 ms (*p* = 0.12).

*Error rates*. An identical ANOVA on arcsine-transformed error rates yielded identical results as response times.

**Implications**

This finding is yet another example of how subtle differences between tasks suggest to the participants different selective usages of features in monitoring – here, to monitor only one feature or several features at a time. A general resource limitation is obviously not responsible for the usage of only one feature during target search at a time, as visual working memory capacity is usually found to be around four items (Luck and Vogel, 1997).

These findings are surprising in light of the assumed resource limitations in this situation. For instance, if working memory was used for the maintenance of the color-search templates, keeping two feature templates active should not have created a cost, as this number of features is well inside the typical resource capacity estimate of (visual) working memory (cf. Luck and Vogel, 1997; Cowan, 2010; but see Oberauer and Hein, 2012). In addition, this is also at variance with what others claim to have observed in a very similar experimental protocol: that participants can search for two colors simultaneously (Kerzel and Witzel, 2019). Also noteworthy, using a similar experimental protocol as Van Moorselaar et al. (2014; cf. Figure 2A), a later study by Hollingworth and Beck (2016) found memory-driven capture also when multiple items were held in working memory, and both these studies were recently replicated, suggesting that both studies yielded robust results and the different outcomes were, thus, suggestive of an impressive flexibility of processing.

In our view, these findings jointly suggest that the observed selectivity could result from flexible cognitive procedures that depend on specific task representations rather than a structural limitation of cognitive resources. If we admit that humans are free to restrict their momentary monitoring focus to only a subset of all possible steering values, thus intentionally creating selectivity, it becomes easy to understand that expected capacity limitations can be violated by self-imposed restrictions. This might occur simply habitually as a consequence of prior experience (for an example, see Box 2).

Here, we discuss two related objections. First, why should participants accept processing costs (here, by searching for a single color at a time) if that could be prevented by a more clever choice of a task representation (here, by searching for two colors at the same time), if not because of a resource limitation forcing them to do so? The answer to this objection is simple: (Some) participants might simply not register the corresponding cost as something that they could prevent by a smarter task representation. For example, the necessity to keep different top-down features apart for the control of other procedures in different contexts (as we have discussed in the example of syllogistic reasoning above) might simply generalize to top-down search for two colors as a default. In line with this possibility, following learning, top-down control settings generalize to transfer tasks in visual search (e.g., Shiffrin and Schneider, 1977; Leber and Egeth, 2006). If participants do not notice the associated costs of this transfer, they would probably not change their task representations. In addition, if participants are generally more familiar with using different features for different purposes in many other situations, this might also create an implicit learning effect that is more difficult to overcome intentionally if that is required or advised (cf. Shiffrin and Schneider, 1977). (Below, we return to this issue).

Secondly, from the perspective of resource theory, selectivity for single features below capacity, as reflected in Büsel et al. (2019), might be particularly surprising (Lavie, 1995, 2005). Lavie (2005), for example, suggests that selectivity for a single feature under simple visual search conditions (e.g., for a single feature) is impossible, as under these conditions sufficient resources are available for the processing of additional input. And yet, this is what humans do: even during visual search for a single feature or while focusing on a single object, they can ignore additional input entirely, even if this is salient (e.g., Eimer and Kiss, 2008; Eitam et al., 2013; Schoeberl et al., 2019).

In fact, theoretically, any decision could always be taken by successively walking through the options at hand, one by one (cf. Kurzban et al., 2013). An interesting prediction that follows from this possibility is that under two-alternative choice response conditions, participants could consistently start with one of these options and test the hypothesis that the conditions for this option are satisfied – for example, that the current sensory input matches the searched-for feature. If one of two options is preferentially monitored first across trials of an experiment and across participants, one should observe a temporal advantage for this option relative to the alternative. In other words, it would be the less preferred, or secondary, option that would suffer from slowing when changing from a condition in which only one of the options is available to a condition in which either of these options can be available in every trial.

Interestingly, this is exactly what has been reported in some experimental situations: for example, if two features – one relevant and one irrelevant – lend themselves to humans’ consistent coding as “positive” or prioritized versus “negative” or less prioritized (Proctor and Cho, 2006), one can find indeed that responses to positive features are faster (Lakens, 2012; Kawai et al., 2020). Take the example of the study by Kawai et al. (2020). Participants were asked to categorize words (e.g., enemy) as positive or negative. In one monochromatic block of trials, these words were all green, in a second monochromatic block, the words were all red, and in a heterochromatic block, red and green words were intermixed and each word was presented in red and green equally often. This was done to understand the origin of the congruence effect between color and affect – here, faster responses to positive words in green and to negative words in red (i.e., in the congruent condition) than to positive words in red and to negative words in green (i.e., in the incongruent condition) (Kuhbandner and Pekrun, 2013). As a consequence of the faster responses to the preferred option (typically the plus pole stimuli, positive words and green words) under choice conditions, a congruence effect (i.e., more efficient performance in congruent than incongruent conditions) based on the similarity versus dissimilarity of the participants’ assigned polarities of two features of a stimulus (here, affect and color) is stronger for the plus pole than for the minus pole. For the plus pole, two positive features (i.e., the positive meaning of the word and its green color) and their polarity congruence (i.e., word meaning and color were both positive, fitting to one another) benefit responses to the congruent stimulus. At the same time, one positive (i.e., the positive meaning of the word) and one negative (i.e., the red color of the word) feature, as well as their polarity incongruence (i.e., word meaning was positive, but color negative, not fitting together) put responses to the incongruent stimulus at a disadvantage. Thus, the congruence effect is substantial. For the minus pole, however, in the congruent condition, two negative features (i.e., negative word meaning and red color) delay responding while their congruence (i.e., the word meaning and the color were both negative, thus fitting together) facilitates responding. Additionally, in the incongruent condition, one positive (i.e., the word meaning) and one negative (i.e., the color red) feature also reflect a mix of accelerating and slowing influences on response speed: facilitation by the positive feature (i.e., word meaning) and slowing of responses by the negative feature (i.e., the red color) as well as the incongruence between the feature polarities (i.e., the positive word meaning and the negative color). Thus, the congruence effect in this case is weaker (cf. Lakens, 2012; Kawai et al., 2020). Importantly, there was no congruence effect in the monochromatic blocks in which the colors did not differ and, thus, participants were not inclined to assign choice-elicited preferences to different colors. This is in line with the decisive nature of the alternative choice options for (1) the preference-dependent reaction time differences (or polarity assignments) and, thus, (2) the congruence effect based on these preferences (the polarity congruence effect). The fact that preferential processing of one color depends on the presence of the alternative color “option” is perfectly in line with the assumed possibility of solving choices by sequentially cycling through the alternative options.

## Dual-Process Theories

So far, we have taken a skeptical stance regarding resource theories by looking at alternative origins of selectivity in terms of procedural control. However, a perhaps even stronger challenge for resource theory are instances of seemingly resource-free processing. Some types of stimulus selection seem to occur even against the human will to concentrate on a task. This is at variance with resource theory (but see Lavie, 1995, 2005, and the discussion further below). These forms of selectivity are interesting, as they are also puzzling from the selection-for-procedures view. The typical “solution” by resource theory has been to assume two types of processing – one depending on limited resources, the other free of resource requirements (e.g., Posner and Snyder, 1975). This is costly, as two rather than one type of processing have to be assumed. Below, we will explain that the functional selection-for-procedures view provides a more parsimonious explanation, showing that seemingly capacity-free processing is often simply an indirect consequence of the way a procedure is controlled and monitored (e.g., Ansorge and Wühr, 2004).

Let us start with typical examples of evidence for two modes of processing, one capacity-limited, depending on resources, and another one resource-free, running independently of capacity limitations (Posner and Snyder, 1975; Tversky and Kahneman, 1983; Petty and Cacioppo, 1986; Sloman, 1996). One famous example of resource-independent processing is “automatic reading” as reflected in the Stroop effect (cf. Stroop, 1935). When having to name the print colors of color words, participants are not able to ignore word meanings, so that an incongruence between word color and word meaning (e.g., the word green written in red), results in slower responses than congruence (e.g., the word blue written in blue) (MacLeod and MacDonald, 2000). It has been emphasized that the opposite is not true: when having to read the words, incongruent colors do not interfere with reading. Hence, it was assumed that reading is practiced to such an extent that it has been automatized and that it can proceed in a resource-free manner. Thus, reading can interfere with naming the word print or font colors (e.g., Posner and Snyder, 1975).

Take the Simon effect as a second example (Simon, 1990; Simon and Craft, 1970; Simon et al., 1970). When humans have to select and discriminate stimuli in their environment, stimulus position affects response efficiency even if the task does not require the processing of stimulus position. For instance, presenting red and green stimuli to the left and the right, and asking participants to discriminate between stimulus colors by left versus right responses, participants are typically faster and, on average, perform more correctly if stimulus and response side correspond to one another than when they do not correspond (e.g., Roswarski and Proctor, 1996): having to press the left key for green and the right key for red stimuli, responses are faster for green stimuli on the left and for red stimuli on the right than for green stimuli on the right and for red stimuli on the left. This Simon effect is very persistent and is also observed for other (e.g., vertical) stimulus-response correspondences (e.g., Stürmer et al., 2002). Originally, it has been interpreted to reflect a dual-process architecture, with a controlled processing route, responsible for the selection of task-dependent responses to the colors, and an automatic processing route, responsible for the automatic activation of response sides or positions by stimulus positions (Kornblum et al., 1990; De Jong et al., 1994; Zorzi and Umiltá, 1995; Zhang et al., 1999).

Let us take peripheral cueing of attention as a third example (Posner et al., 1980). During visual search for a target, presenting a peripheral cue prior to the target facilitates target search if the target is presented at the same position as the preceding cue (valid or cued condition), but interferes with target search if the target is presented away from the preceding cue (invalid or uncued condition). Originally, it was believed that this is due to automatic capture of attention by the peripheral cue, such that attention needed to be shifted to the target in uncued but not in cued conditions. Automaticity was assumed, as the cueing effect of peripheral cues (i.e., the advantage for targets at cued vs. uncued locations) was even found for cues that were not predictive of the upcoming target location. Furthermore, a short interval between cue and target and, thus, little time for cue processing enhanced the effect (Jonides, 1981; Müller and Rabbitt, 1989). In fact, participants could not suppress peripheral cues even when asked to do so (Jonides, 1981).

Later research, however, has proven all these initial interpretations as too simplistic. In all of these classic empirical cases for capacity-free selection of information, procedural control turned out to be responsible for the “automatic effects,” too. Let us first look at the Stroop effect. Some studies noted that the Stroop effect is strongest if the color and the word belonged to the same object, implying that the word was selected inadvertently together with the color of an object, but not or less so if the word and the color were independent objects (Besner et al., 1997; Wühr and Frings, 2008). This observation suggests that in these situations, humans do not necessarily read a word automatically. Instead, the task of attending to the color of an object entails that the object carrying the color would also be processed to some extent. According to this interpretation, the functional task demands of having to select colors would be responsible for the inadvertent selection of the word meanings, too. However, one could argue that it is also possible that irrelevant words would be automatically read but that it is easier to suppress their influences or to actively filter out the words if they are represented in or as a different object (cf. Wühr and Frings, 2008). And yet, more or less Stroop interference depending on the presence of words and colors in the same objects is not the only evidence in favor of a functional origin of the selection of the word meanings. In a dramatic demonstration of the dependence of the Stroop effect on procedural control, Durgin (2000) reversed the Stroop effect simply by changing the response requirements (see Figure 2C). He asked participants to point to color patches corresponding to the word meanings rather than to utter the color names, thereby increasing the fit between irrelevant word colors and required responses (and decreasing the fit between word meanings and responses). As a consequence, Durgin observed that irrelevant but incongruent word colors interfered with word reading and that irrelevant word meaning’s interference on discriminating between word colors was almost non-existent. These findings show that response requirements and the resulting match of stimuli to the responses differed in a way as to either facilitate color or word processing. In response to such findings, it is possible to identify different dimensions of potential overlap between stimuli and responses, all of which could interactively or additively determine the resulting net compatibility or correspondence effects based on automatic selection of stimulus features (cf. Kornblum et al., 1990). Critically, however, this description assumes that both stimuli and responses would be somehow discriminated from one another irrespective of the task at hand. Thus, this position leaves open as to why it would be possible to represent responses themselves differently.

The critical involvement of flexible representations of the responses (or, more generally, of the intended outcomes of a procedure) for interference by seemingly irrelevant feature or stimulus selection that is only predicted by the functional view and not by any kind of resource-free selection interfering with resource-demanding processing, was demonstrated in the Simon effect (Hommel, 1993; Ansorge and Wühr, 2004; Wühr and Ansorge, 2007). Consider the study of Hommel (1993). Participants had to discriminate the pitch of sounds presented from either the left or the right, responding left for low pitches and right for high pitches. Any button press additionally caused a light to turn on in the opposite hemifield. Crucially, in one condition, participants were instructed to respond with a button press, while in the other, they were instructed to turn on the light.

As a consequence, Hommel (1993) observed inverted Simon effects in the conditions in which lights had to be turned on: now stimuli on the right facilitated left-key presses and stimuli on the left facilitated right-key presses. Hommel (1993) reasoned that this was due to the flexible representation of the required responses in terms of their different potential sensory features (or, to be exact, sensory features of their effects or outcomes), such as the visually perceived or felt position of the response buttons (e.g., in a more traditional stimulus-response instruction, where the task was to press buttons) or as the visually perceived light positions (where the task was to turn on lights). As even the perceived light positions reliably discriminated between the required responses and, thus, could have been used to monitor the responses, participants included light positions in their response representations even prior to stimulus processing and, hence, a correspondence effect based on the intended and monitored responses resulted (or response effects, cf. Stoet and Hommel, 1999; Kunde, 2001; Ansorge, 2002).

That the flexible nature of the response representations rather than some pre-existing correspondence between stimuli and responses accounted for the Simon effect was substantiated by research of Ansorge and Wühr (2004). In each trial of their experiments (see Figure 2B), these authors presented a visual stimulus at one of four different positions, located above or below, left or right of the screen center. Critically, stimulus colors (red vs. green) were to be discriminated by two-alternative forced-choice responses varying on both axes – horizontal (left or right) and vertical (above or below), but responses differed from one another only on one of these axes. For instance, red required pressing a button to the left and above of a home key, while green required pressing a button to the left and below of the home key, meaning that the vertical but not the horizontal axis discriminated between the responses. In this way, participants’ functional response representations were gauged to include the discriminative axis positions (e.g., in the example above on the vertical axis), but automatic effects of stimulus-response correspondence were possible for both axes. For instance, in the example above, stimuli on the vertical and on the horizontal axes could have exerted stimulus-response correspondence effects, as stimuli varied on both axes and both of these axes were part of a required response. In line with a flexible and functional perspective of response representations, however, the Simon effect was restricted to the response-discriminating stimulus positions. It was absent for the non-discriminating axis. For example, if red required a response to the left and above and green required a response to the left and below, participants were faster to respond to green stimuli below than above fixation, but their response was not affected by whether the green stimuli were presented left or right of fixation. This was the case, although only half of the green stimuli (the ones on the left) would have been presented on a side corresponding to the side of the required responses. Hence, only discriminative response features created a Simon effect, a finding much more in line with a functional view and flexible response representations (cf. Hommel, 2004) than with a view that assumes that somehow stimuli unfold their effects in a rigid and task-independent two-process architecture (e.g., Kornblum et al., 1990).

The same conclusion that has been drawn regarding the Stroop effect and the Simon effect – that the seemingly automatic selection of visual information depended on subtle forms of procedural control, has been reached for peripheral cueing. Specifically, in their contingent involuntary orienting hypothesis, Folk et al. (1992) tested if peripheral cues preceding targets at potential target locations might have captured participants’ attention via matching the attentional control settings set up for the targets. These authors used two types of peripheral cues: abrupt onset cues, that is, a single white cue presented at one of several target positions, and color cues, that is, a single differently colored cue (e.g., a red cue) presented at one of several potential target positions along with color non-singletons (e.g., green non-singletons) at all other potential cueing (and target) positions. According to known bottom-up theories, all of these cues were salient – that is, they differed by strong local feature differences (e.g., in color) from their surroundings, and all of these cues should have therefore been in a position to capture attention automatically, in a stimulus-driven way (cf. Theeuwes, 1992; Nothdurft, 1993; Itti et al., 1998). To test if these cues captured attention automatically, Folk and Remington (1998) used two different blocked search conditions, matching the two possible cue-types in turn: targets were either abrupt-onset singletons (i.e., the single stimulus with an abrupt onset in the target display) in one blocked condition; or targets were color singletons (i.e., the single stimulus standing out by its odd color among homogeneously colored non-singletons of a different color). These authors found that color cues captured attention during search for color-defined targets but not during search for abrupt-onset targets and that abrupt-onset cues captured attention during search for abrupt-onset targets but not during search for color-defined targets. Later research confirmed that even the cue’s color had to be similar to the searched-for color of the target (Folk and Remington, 1998). These results support the top-down contingency of the involuntary capture of attention by the cue on the cue’s match to the top-down search settings (or the attentional control sets). Importantly, the evidence cannot be better explained by inter-trial priming of color (here, from a target in a preceding Trial *N*−1 to a cue in the current Trial *N*) and it is not better explained by quick capture of attention by just any salient cue – be it a top-down matching or a non-matching cue – and subsequent quick inhibition of capture by the non-matching cue only (cf. Ansorge and Horstmann, 2007; Eimer and Kiss, 2008; for a meta-analysis and review, see Büsel et al., 2020). For example, during search for two potential target colors, when both color-singleton cue and color non-singletons had a top-down matching color, there was no cueing effect, as all stimuli – singleton cue and non-singletons – matched the top-down control settings and, thus, attention was not captured to only the single more salient position of the singleton cue (Schoeberl et al., 2019).

In this context, it is worth noting that one particular variant of dual-process theories – namely load theory (cf. Lavie, 1995, 2005) was also not supported by the findings. According to load theory, stimulus-driven capture of attention as a form of selectivity prevails under conditions of low perceptual demands, whereas high perceptual demands would prevent stimulus-driven capture of attention. However, a salient but non-matching abrupt-onset singleton cue does not even capture attention when presented under very slightly perceptually demanding conditions: if presented alone – without concomitant competing distractors (Goller et al., 2016, 2020b). This failure of stimulus-driven capture of attention is evident in a continuous tracking of the cue’s capture of attention by N2pc (e.g., Arnott et al., 2001; Goller et al., 2020b), an event-related potential that reflects shifts of attention to the left or the right (cf. Luck and Hillyard, 1994). In this context, the N2pc reflects more negative activity on the side contralateral than ipsilateral to an attended-to stimulus. The N2pc starts at about 200 ms post-stimulus and allows to continuously track the capture of attention with millisecond resolution, right from stimulus onset onward. Thus, it can be used to measure attention capture elicited by the cue itself, without having to rely on overt responses to the target (as would be the case for the cueing effect in target reaction times). Thus, the lack of any cue-elicited N2pc is particularly convincing evidence against any automatic capture of attention under conditions of slight or low perceptual demands (cf. Eimer and Kiss, 2008; Goller et al., 2020b).

In conclusion, many instances of seemingly resource-free processing can be more elegantly traced to subtle side effects of procedural control rather than a dual-process framework. In contrast, the only resort for explaining these effects from the perspective of resource theory is to allow a separate category of resource-free processes, as, otherwise, it would be hard to understand why humans would spend some of their precious cognitive resources on these seemingly irrelevant forms of selection. To note, participants might also avoid investing even more of their limited resources into active suppression of interfering stimuli. This, however, presupposes that something like resource-free processing existed in the first place. This assumption, we believe, is at least not always warranted given the subtle task-dependencies that we identified.

## Neural Resources

An obvious argument in favor of some form of resource limitation comes from neurophysiological data. Ultimately, the number of neurons in the human nervous system is finite and so is their upper limit of information processing. Whether, over the course of evolution, procedural demands shaped physiology or physiology determined cognitive abilities constitutes somewhat of a hen-and-egg problem. Interestingly, recent evidence on perceptual, attentional, or working-memory related limitations does fit exceptionally well with our proposed limitations via functional procedural control. In particular, several recent studies have demonstrated that environmental locations, objects or features seem to be ‘sampled’ by the brain in discrete steps rather than in a continuous fashion (for a review, see VanRullen, 2016). This sampling process likely originates from the ubiquitous rhythmic neural activity, which constitutes alternating phases of facilitated and suppressed information processing. Crucially, when this sampling process is directed to more than one location, feature or object at a time, the respective dimensions are sampled serially in alternation, rather than in parallel and at the same time. For instance, participants simultaneously monitoring two spatial locations for visual targets showed rhythmic fluctuations in target detection between 4 and 10 Hz. In line with a limited resource, temporal fluctuation profiles for the two locations were in anti-phase, suggesting that selection from two locations had to alternate between locations (Landau and Fries, 2012). Recently, we demonstrated a similar mechanism also for target-relevant templates held in working memory (Pomper and Ansorge, accepted): detection performance for targets corresponding to internally held templates was not continuous but fluctuated rhythmically over time. Importantly, performance fluctuations for two simultaneously held templates were in anti-phase, suggesting that a single working memory template is prioritized at any point in time. Critically, however, in our view, such selectivity does not imply that the ultimate origin of the alternating performance fluctuations was a limited neurophysiological resource. Instead, rhythmically alternating fluctuations could simply illustrate how monitoring of either of several locations, objects, or features at a time is realized at the physiological level. Concerning locations, this is particularly obvious, as even looking at a location – that is, the most natural response in a perceptual task – would require that we focus on one location at a time (cf. Rizzolatti et al., 1987), and what would be more natural than to rhythmically switch between single locations if more than one needs monitoring? In other words, an intention to preferentially monitor only one location at a time might simply be one way of how the task can be routinely solved at all. Thus, this intention for procedural control could be the ultimate reason behind this behavior, and oscillations may simply be one way in which brain processing could be used to fulfill these forms of procedural control.

## Discussion

Having argued for care in interpreting selectivity as reflecting structural capacity limits rather than functional selection imposed by top-down control of procedures, we want to emphasize that we do not want to question the possibility of limited resources or their counterpart – automatic processing – altogether. Certainly, some tasks are so difficult that they exceed limited human processing capacity while being easily performed by modern-day computers. Instead, our review highlighted examples of hyper selectivity in fairly simple tasks to caution against over-interpreting just any selectivity as evidence of an underlying structural resource limitation.

In addition, we took a skeptical stance toward dual-process theories as an explanation of several instances of seeming automatic or resource-free processing. Instead, we suggest taking a functional perspective and understanding these processes in terms of the top-down control of procedures. However, we believe that not all processes can be explained easily as forms of inadvertent processing through top-down control of procedures. For instance, flicker singletons with a flicker frequency deviating from that of their surrounding stimuli seem to capture attention in a truly bottom-up, automatic, or resource-free way (Cass et al., 2011; Stolte and Ansorge, 2021).

Another issue altogether are the types of learning-dependent automatic selection (cf. Shiffrin and Schneider, 1977). Highly trained forms of selection in the pursuit of persisting task demands are very likely under the selection-for-procedures view, as some of the types of controlled procedures that humans perform are very frequent in the everyday world. Again, think of looking at relevant locations as a strategy to support perception. These forms of selections may also spill over or generalize to situations in which they are not optimal or at least not necessary (Luchins, 1942; Goller et al., 2020a). Take the example of Goller et al. (2020a). These authors used Korean and German speakers in a test of language-induced tendencies to select visual inputs. Only the Korean language but not German (or English for that matter) strictly requires choosing a verb appropriate to discriminate tight- versus loose-fit relations between objects. Hence, Korean speakers should have practiced this particular procedure of selecting the corresponding visual information for an appropriate verbal description much more often than German speakers. In line with this hypothesis, even in a non-linguistic visual search for color-defined targets, Korean speakers showed a higher sensitivity for selecting a “fit singleton” than German speakers. Specifically, during search for a red target, presenting a differently colored fit singleton (e.g., a combination of loose cylinder around a piston presented among fit non-singletons, e.g., combinations of tightly fitting cylinders around pistons at all other positions) away from the target captured Korean speakers’ but not German speakers’ attention. This was evident in longer search times with interfering fit-singleton distractor than in a baseline condition without fit-singleton distractor (Experiments 4 and 5 of Goller et al., 2020a). This effect did not reflect simply more automatic attention capture among the Korean speakers, as capture and interference by a color singleton was the same for Korean and German speakers. Rather, it, reflected a generalization of a practice-dependent selection in the service of procedural control (here, depending on the practice with the language that one speaks) to a non-linguistic color-search task (cf. Baier and Ansorge, 2019). We cannot say if this selection reflected a form of more change-resistant gradual learning (cf. Shiffrin and Schneider, 1977) or a form of “Einstellung effect” (cf. Luchins, 1942), but we acknowledge the existence of these forms of “long-term procedural selectivity” that is not due to the task representation set up for procedural control in a current situation. Importantly, both of these factors are founded in the control of procedures rather than being due to resource limitations.

## Conclusion

Humans are literally born in the saddle. They are born into a physically extended world, including their own bodies, which evolves over time. They have to control their bodily actions for successful coordination within a dynamically changing environment. Out of these constraints arises a necessity to select information appropriate to coordinated action in the temporally evolving spatial surroundings. This is a major reason for selectivity in processing, the consequences of which are often dismissed too easily as mere resource limitations. Therefore, in the current review, we have taken a skeptical stance toward the resource view, as selectivity can express how humans exert control over procedures in general, be these overt actions or covert processing. Importantly, this selection-for-procedures view is a functional, not a structural perspective. It emphasizes that selectivity is a benefit for information processing, not a deficit of it. Our view comes close to existing theories, such as Janczyk and Kunde’s (2020) conclusion that anticipated action effects could explain psychological refractory period effects: what I as an agent expect to happen as a consequence of my processing or actions is responsible for the necessity to either deal with one task or the other at a time. However, in contrast to these authors, we do not think that the resulting bottleneck is of a structural nature – that is, the anticipation of procedural consequences does not draw on a limited resource that could be used for one or the other task. Instead, we think that a human’s choice of the anticipated and monitored procedural consequences is her or his way to flexibly control her procedures itself.

## Data Availability Statement

The original contributions generated for this study are included in the article/supplementary material, further inquiries can be directed to the corresponding author.

## Ethics Statement

Ethical review and approval was not required for the study on human participants in accordance with the local legislation and institutional requirements. The patients/participants provided their written informed consent to participate in this study.

## Author Contributions

UA, CB, MF, DG, MG, UP, MS, RS, and CV planned the outline and content of the present article. UA drafted a first version of the manuscript, with the exceptions of Box 2 and of Figure 2, the first versions of which were crafted by CB and CV, respectively. All authors commented upon and revised the manuscript several times.

## Conflict of Interest

The authors declare that the research was conducted in the absence of any commercial or financial relationships that could be construed as a potential conflict of interest.

## Publisher’s Note

All claims expressed in this article are solely those of the authors and do not necessarily represent those of their affiliated organizations, or those of the publisher, the editors and the reviewers. Any product that may be evaluated in this article, or claim that may be made by its manufacturer, is not guaranteed or endorsed by the publisher.
